# The calcium channel TRPV6 is a novel regulator of RANKL‐induced osteoclastic differentiation and bone absorption activity through the IGF–PI3K–AKT pathway

**DOI:** 10.1111/cpr.12955

**Published:** 2020-11-07

**Authors:** Jun Ma, Lei Zhu, Zhibin Zhou, Tengfei Song, Lei Yang, Xu Yan, Aimin chen, Tian Wen Ye

**Affiliations:** ^1^ Department of Orthopedic Trauma Surgery Changzheng Hospital The Second Military Medical University Shanghai China; ^2^ Department of Health Statistics The Second Military Medical University Shanghai China; ^3^ Department of Orthopedic Surgery General Hospital of Northern Theater Command Shenyang China; ^4^ Department of Orthopedic Surgery The 2nd affiliated Hospital of Wenzhou Medical University Wenzhou China; ^5^ Department of Orthopedic Surgery Naval Characteristic Medical Center The Second Military Medical University Shanghai China

**Keywords:** IGF‐PI3K‐AKT, osteoclast, osteoporosis, TRPV6

## Abstract

**Objectives:**

Calcium ion signals are important for osteoclast differentiation. Transient receptor potential vanilloid 6 (TRPV6) is a regulator of bone homeostasis. However, it was unclear whether TRPV6 was involved in osteoclast formation. Therefore, the aim of this study was to evaluate the role of TPRV6 in bone metabolism and to clarify its regulatory role in osteoclasts at the cellular level.

**Materials and methods:**

Bone structure and histological changes in *Trpv6* knockout mice were examined using micro‐computed tomography and histological analyses. To investigate the effects of Trpv6 on osteoclast function, we silenced or overexpressed *Trpv6* in osteoclasts via lentivirus transfection, respectively. Osteoclast differentiation and bone resorption viability were measured by tartrate‐resistant acid phosphatase (TRAP) staining and pit formation assays. The expression of osteoclast marker genes, including *cathepsin k, DC‐STAMP, Atp6v0d2* and *TRAP,* was measured by qRT‐PCR. Cell immunofluorescence and Western blotting were applied to explore the mechanisms by which the IGF‐PI3K‐AKT pathway was involved in the regulation of osteoclast formation and bone resorption by Trpv6.

**Results:**

We found that knockout of *Trpv6* induced osteoporosis and enhanced bone resorption in mice, but did not affect bone formation. Further studies showed that Trpv6, which was distributed on the cell membrane of osteoclasts, acted as a negative regulator for osteoclast differentiation and function. Mechanistically, Trpv6 suppressed osteoclastogenesis by decreasing the ratios of phosphoprotein/total protein in the IGF–PI3K–AKT signalling pathway. Blocking of the IGF–PI3K–AKT pathway significantly alleviated the inhibitory effect of Trpv6 on osteoclasts formation.

**Conclusions:**

Our study confirmed the important role of Trpv6 in bone metabolism and clarified its regulatory role in osteoclasts at the cellular level. Taken together, this study may inspire a new strategy for the treatment of osteoporosis.

## Graphical abstract

The transient receptor potential vanilloid 6 (TRPV6) was a critical negative regulator in RANKL‐induced osteoclast differentiation and bone resorption activity. TRPV6 negatively regulates osteoclast formation and bone resorption by inhibiting the IGF/PI3K/AKT signalling pathway.

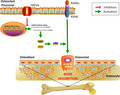

## 1 BACKGROUND

Osteoporosis is a common global metabolic bone disease characterized by decreased bone mineral density and bone mass.^1^, ^2^ Bones exist in a dynamic balance, which mainly includes two parts: bone formation and bone resorption. Osteoporosis occurs when bone resorption is greater than bone formation, thereby decreasing bone mass.^3^, ^4^ Decreasing bone mass is caused by the abnormal differentiation and proliferation of osteoclasts that trigger bone resorption, so osteoclasts are considered to be a target cell type for treating osteoporosis.^5^, ^6^, ^7^ Despite the regulation of osteoclast formation and function by numerous cytokines and hormones, the macrophage colony‐stimulating factor (M‐CSF) and receptor activator of NF‐κB ligand (RANKL) are the most critical molecules for osteoclastogenesis.^8^, ^9^, ^10^, ^11^ Calcium ion (Ca^2+^) channels are also essential for osteoclast differentiation.^12^ Extensive studies have indicated that RANKL induces oscillatory changes in intracellular Ca^2+^ concentrations and activates nuclear factor of activated T cells c1 (NFATc1), resulting in osteoclast‐specific gene transcription to induce osteoclast differentiation.^13^, ^14^ However, the exact calcium signals involved in RANKL‐induced osteoclast differentiation need further clarification.

The transient receptor potential vanilloid (TRPV) protein family is a family of calcium transport proteins of which only TRPV5/6 are highly selective calcium channels.^15^, ^16^, ^17^ Recent studies have found that TRPV6 is an important calcium channel involved in the regulation of bone metabolism.^18^, ^19^, ^20^, ^21^ However, the specific molecular mechanism of how TRPV6 is involved in bone metabolism remains elusive and needs to be further explored.

The IGF signalling pathway widely exists in bone tissue and is central to the regulation of bone metabolism.^22^, ^23^ Many studies have indicated that IGF1R and IGF1 proteins are expressed in osteoclasts and that IGF1R is involved in osteoclast differentiation.^24^, ^25^, ^26^ However, whether the IGF signalling pathway is involved in the regulation of osteoclast formation and bone resorption by TRPV6 remains unclear.

In this study, we investigated the mechanism of TRPV6 in osteoclast formation and bone resorption activity. Our in vivo studies revealed severe osteoporosis in *Trpv6* knockout mice. Inactivation of Trpv6 did not affect bone formation but significantly enhanced bone resorption. Furthermore, we found that Trpv6 negatively regulated osteoclast differentiation and bone resorption by inhibiting the IGFIR–PI3K–AKT pathway. Taken together, our study suggests that Trpv6 is a potential therapeutic target for treating osteoporosis.

## 2 MATERIALS AND METHODS

### 2.1 Mice transgenic lines and mice care

Eight‐week‐old *Trpv6* knockout mice and wild‐type mice (from the Laboratory Animal Center of the Second Military Medical University) were used in this study.^27^ All of the mice were housed in rearing cages (five mice per cage) and kept under standard laboratory conditions (12‐hour light‐dark cycle; 25°C). Experiments were approved by the Medical Ethics Committee of the Second Military Medical University.

### 2.2 Skeletal phenotyping

To assess bone density and trabecular micro‐architecture, the distal femurs were taken to scan using micro‐computed tomographic imaging (SkyScan). The scanning analysis area was the trabecular portion of the proximal femoral growth plate from 2 mm downward. Micro‐architectural parameters include bone mineral density (BMD), trabecular number (Tb.N) and Bone Volume/Total Volume (BV/TV).

### 2.3 Histological analyses

Harvested femurs were fixed in 4% paraformaldehyde solution (Aladdin) and decalcified in EDTA buffered saline solution (Aladdin). Sagittal tissue sections (10 μm thick) were prepared for immunohistochemistry. Haematoxylin‐eosin staining, Masson's trichrome staining and TRAP staining were performed afterwards to assess the histology.

### 2.4 Dynamic bone formation

Mice femurs were dehydrated with different concentrations of alcohol (70%‐100%) and embedded in methyl‐methacrylate (MMA; Sigma‐Aldrich). The bone tissue was then cut and polished to a thickness of about 10 mm. The sections were imaged using a fluorescence microscope (Carl Zeiss). The mineral apposition rate (MAR) was calculated using histomorphometric data.

### 2.5 Measurements of plasma ALP and CTX‐1

Plasma ALP and CTX‐1 concentrations were determined using ELISA kits (Mlbio). After the mice were sacrificed, the blood was extracted from the cardiac cavity via syringe and centrifuged for 5 minutes (2000 rpm, 4℃). The supernatants were incubated for 2 hours on microporous plates. Next, an equivalent volume of anti‐ALP or anti‐CTX‐1 primary antibody was added to each sample and incubated for another 1 hour. This was followed by a 30 minutes incubation with secondary antibodies. Finally, a microplate reader was used to detect absorbance values at 450 nm.

### 2.6 Cell culture

Bone marrow cells were isolated from the femur and tibiae of C57BL/6 mice or *Trpv6* knockout mice in the C57BL/6 background. Cells were cultured in a‐MEM medium (Invitrogen) supplemented with 10% foetal bovine serum (FBS), 1% penicillin‐streptomycin and M‐CSF (Peprotech) for 1 day. Non‐adherent cells were harvested and cultured in medium containing M‐CSF (50 ng/mL) for 3 days. Then, the adherent cells, which were preosteoclasts, were collected. Next, a‐MEM medium containing M‐CSF (30 ng/mL) and RANKL (50 ng/mL) (Peprotech) was added and allowed to incubate for 7 days to generate mature osteoclasts.

### 2.7 Tartrate‐resistant acid phosphatase staining

TRAP staining is the most common staining method to identify mature osteoclasts. A leukocyte acid phosphatase kit (Sigma) was used to fix and stain the cells according to the manufacturer's instructions. Mature osteoclasts (with more than three TRAP‐positive nuclei) appeared dark red and were counted using an optical microscope.

### 2.8 Pit formation assay

Pit formation assays were used to assess bone resorption activity. Bovine cortical bone was polished into thin slices (200 µm thick). The bone slices were placed into a 24‐well plate. Cells were inoculated into the bone slices and induced by M‐CSF and RANKL. Seven days later, the sections were placed in sterile water and washed using ultrasonication for 10 minutes to dislodge the cells. This was followed by staining with toluidine blue for 5 minutes. The percentage of pit areas was quantified using ImageJ.

### 2.9 Cell immunofluorescence

Bone marrow macrophage cells (BMMs) were induced by M‐CSF and RANKL for 7 days. Then, the culture medium was washed with PBS and the cells were fixed with 4% paraformaldehyde for 20 minutes. Afterwards, 0.2% Triton X‐100 was applied for 5 minutes to permeabilize the cells. After washing away the permeabilizing solution with PBS, the permeable cells were blocked with 5% BSA for 30 minutes. Additionally, the primary antibodies (anti‐TRPV6, anti‐IGFIR or anti‐p‐AKT) were added and incubated with the cells overnight at 4°C. PBS was used to wash off the primary antibodies and corresponding secondary antibodies for 30 minutes at 37°C. After the secondary antibody was washed away, the cells were finally fixed with an anti‐fading fixing medium. Fluorescence was observed by confocal laser scanning microscopy (Leica).

### 2.10 Lentiviral transduction and oligonucleotide transfection

Osteoclast precursors were seeded into 6‐well plates at a density of 2 × 10^5,^ and transfection began when the cell fusion rate reached approximately 60%. Cells were infected with TRPV6 shRNA lentiviral particles or TRPV6 lentiviral activation particles (Santa Cruz) separately for 24 hours. The culture medium was then replaced with a normal osteoclast induction medium.

### 2.11 Quantification of mRNA and qPCR

Total RNA was isolated from cells using the TRIzol reagent (Takara Biotechnology), and the first‐strand cDNA was synthesized from RNA via the miScript Reverse Transcription Kit (Takara) according to the manufacturer's protocols. qRT‐PCR was carried out with the SYBR Green PCR kit (Takara Biotechnology), and sequence detection was performed using an ABI 7500 Sequencing Detection System (Applied Biosystems). β‐actin was used as the housekeeping gene. The primers used for expression analysis are listed in Table 1.

**TABLE1 1:** Primers for qRT‐PCR analysis

Genes	Orientation	Sequence (5′ to 3′)	NM transcript numbers
TRPV6	Forward Reverse	CCCAAGCTTATTTTACTGAATTCT CGGGGTACCCTAGTAGGCCCAG	NM_022413
TRPV5	Forward Reverse	ATGGGGGCTAAAACTCCTTGG CCTCTTTGCCGGAAGTCACA	NM_001007572
TRPV2	Forward Reverse	AGCCATTCCCTCATCAAAAG AGCCAGCTCACCCATACC	NM_001382490
TRPV4	Forward Reverse	CGTCCAAACCTGCGAATGAAGTTC CCTCCATCTCTTGTTGTCACTGG	NM_022017
CathepsinK	Forward Reverse	GAAGAAGACTCACCAGAAGCAG TCCAGGTTATGGGCAGAGATT	NM_007802
TRAP	Forward Reverse	CACTCCCACCCTGAGATTTGT CATCGTCTGCACGGTTCTG	NM_001355189
Atp6v0d2	Forward Reverse	TGCGGCAGGCTCTATCCAGAGG CCACTGCCACCGACAGCGTC	NM_175406
DC‐STAMP	Forward Reverse	GGGGACTTATGTGTTTCCACG ACAAAGCAACAGACTCCCAAAT	NM_029422
IGF1R	Forward Reverse	ACCGGGATCTCATCAGCTTCAC TCCTTGTTCGGAGGCAGGTC	NM_010513
IGFBP1	Forward Reverse	TACTATCTACTCAGAAAGTCGTGAC ACACATATATAAAATGGTGTGCTCC	NM_008341
β‐actin	Forward Reverse	GGCTGTATTCCCCTCCATCG CCAGTTGGTAACAATGCCATGT	NM_173979

### 2.12 Western blot analysis

Proteins from osteoclast lysates were introduced to 10% SDS‐PAGE (Coring) and were subsequently electroblotted onto a polyvinylidene fluoride membrane. The membrane was blocked with 5% fat‐free milk and incubated with appropriate antibodies (all from Santa Cruz Biotechnology). Chemiluminescence reagents (Thermo Scientific) were used to detected antigen‐antibody complexes. ImageJ was used to quantify the protein levels from immunoblots.

### 2.13 Statistical analysis

Data are shown as mean ± SD. SPSS24.0 statistical software was used for statistical analysis. Two‐tailed non‐paired Student's *t* tests and one‐way ANOVAs were used to compare differences. Differences were considered significant if *P* < .05, and a high‐level of significance was *P* < .01.

## 3 RESULTS

### 3.1 Knockout of *Trpv6* induces osteoporosis in mice

**FIGURE 1:**
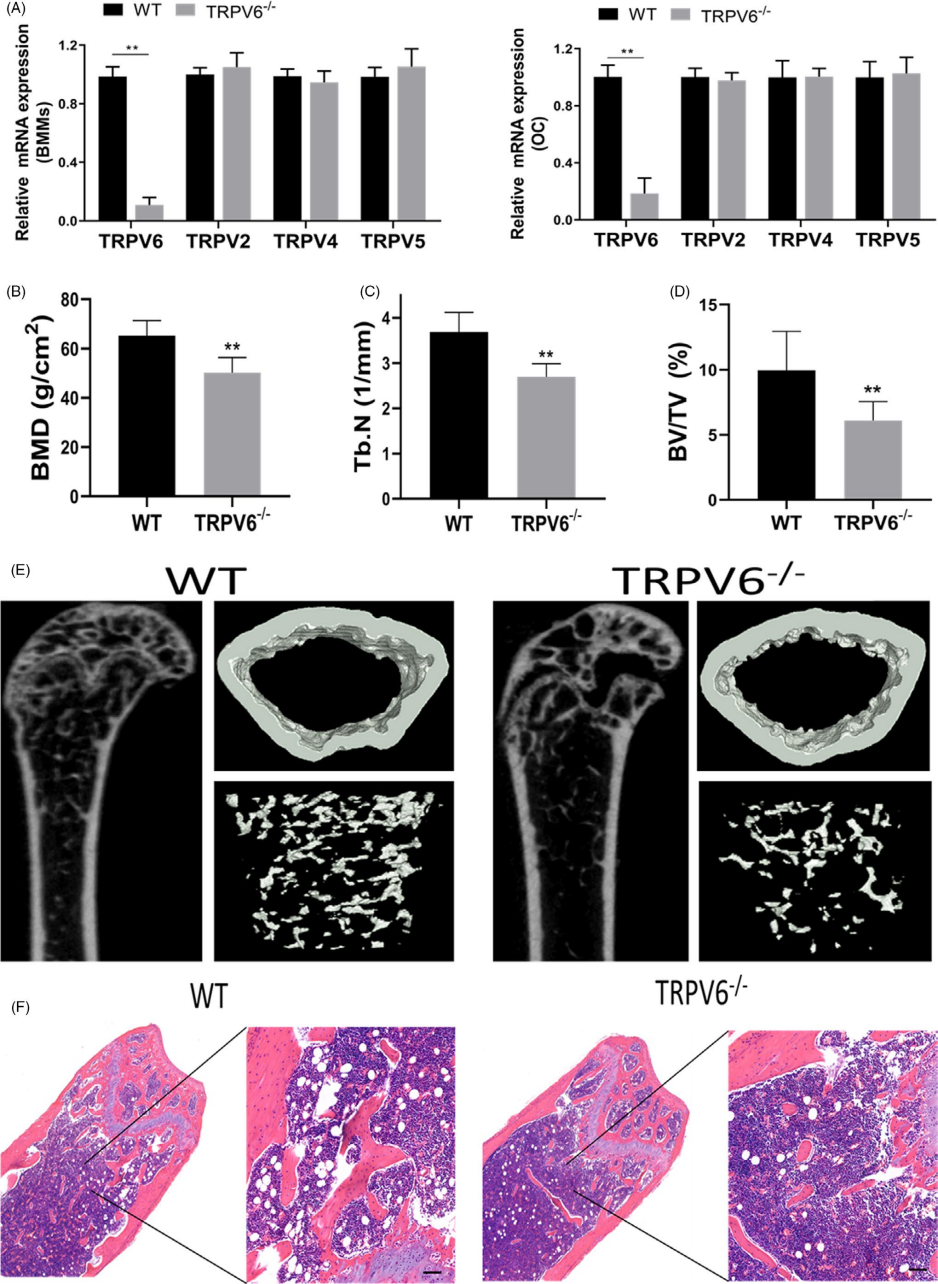
Knockout of Trpv6 induced osteoporosis in mice. (A) qRT‐PCR analysis of Trpv2, Trpv4, Trpv5 and Trpv6 expression in BMMs and OCP from Trpv6−/− mice and WT mice. Summarized data showed that Trpv6 expression was significantly decreased in BMMs from Trpv6‐/‐ mice. n = 3, **P < .01. Trpv2, Trpv4 and Trpv5 expression levels were not significantly changed in BMMs and OCP derived from Trpv6‐/‐ mice and WT mice. n = 3. (B) BMD at the distal end of the intact femurs of each experimental group. n = 5, **P < .01. (C) Tb.N in the distal end of the intact femurs of each experimental group. n = 5, **P < .01. (D) BV/TV at the distal end of the intact femurs of each experimental group. n = 5, **P < .01. (E) Representative figures of micro‐CT analysis of the distal end of intact femurs from Trpv6−/− and WT mice. (F) Haematoxylin‐eosin staining was performed to identify histological structures at the distal end of the intact femurs of Trpv6−/− and WT mice. Scale bars are 50 μm

*Trpv6* knockout mice were used in this study to investigate the biological role of Trpv6 in bone metabolism. To confirm whether the Trpv6‐depleted mouse line was successfully generated, we compared the transcription levels of *Trpv6* in bone marrow monocytes and osteoclast precursors from wild‐type and *Trpv6^−/−^* mice. We found that the expression of *Trpv6* was significantly depleted in *Trpv6^−/−^* mice. Furthermore, there was no significant difference in the expression levels of *Trpv2*, *Trpv4* and *Trpv5* in bone marrow monocytes and osteoclast precursors in *Trpv6^−/−^* mice and WT mice (Figure 1A). At 12 weeks old, mice were euthanized and the femur cancellous bone was analysed by micro‐CT. The results revealed that *Trpv6^−/−^* mice had remarkably decreased BMD (Figure 1B). Analysis of the trabecular structure revealed that the Tb.N and BV/TV were significantly lower in *Trpv6^−/−^* mice compared to WT mice (Figure 1C‐D). Representative μCT images of trabecular bones are presented in Figure 1E, which is consistent with the results of HE staining (Figure 1F).

### 3.2 In *Trpv6* knockout mice, bone absorption was enhanced and bone formation remained unchanged

**FIGURE 2:**
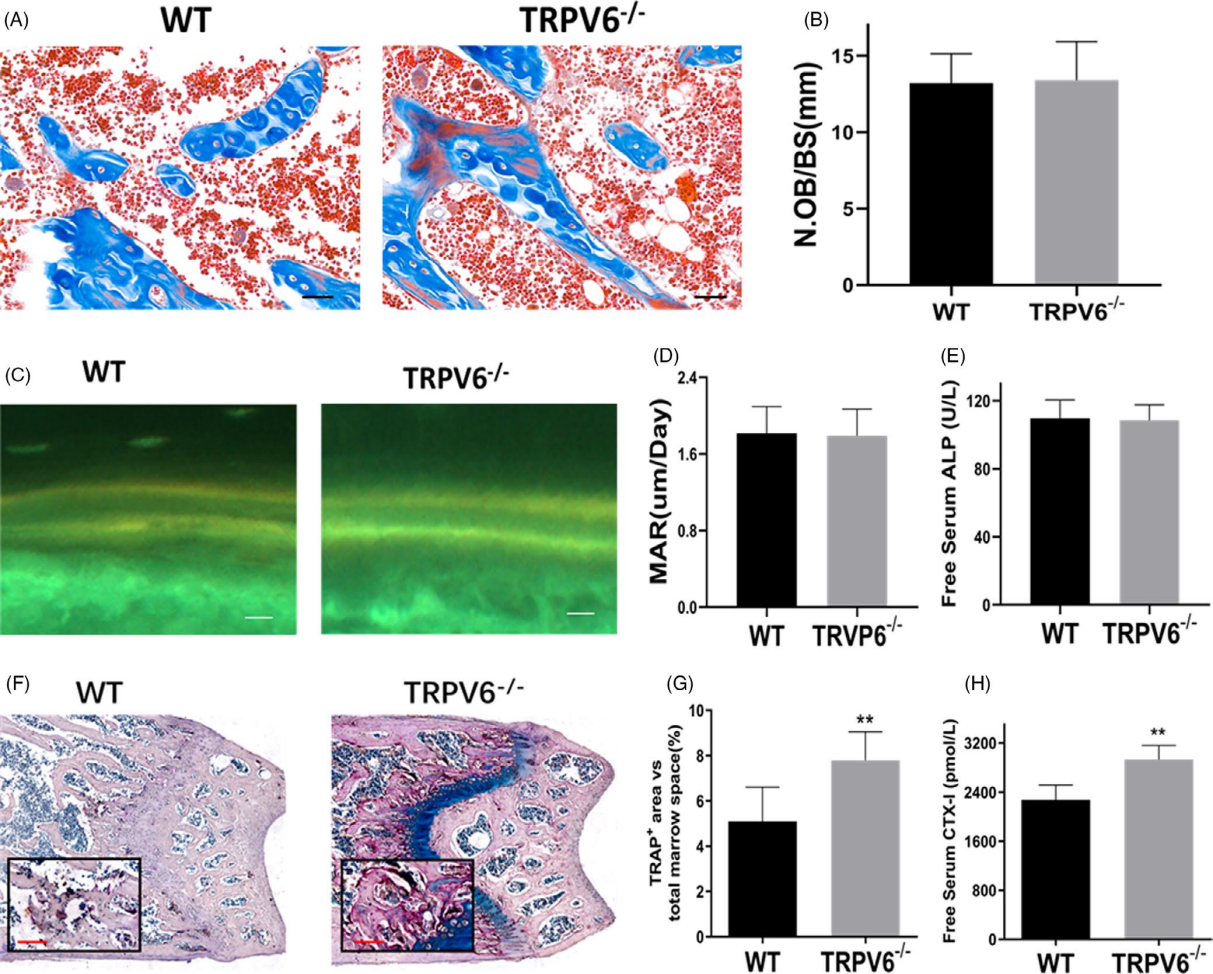
In Trpv6 knockout mice, bone absorption was enhanced and bone formation remained unchanged (A) Histological sections of femurs were subjected to Masson's trichrome staining. Scale bars are 50 μm. (B) Quantitative analysis of osteoclast number/bone surface (N.Ob/BS). n = 5, **P < .01. (C) Tetracycline labelling was observed by fluorescence light microscopy in the slices of the tibia of each experimental group. Scale bars are 50 μm. (D) Quantitative analysis of mineral apposition rate. n = 5, **P < .01. (E) Serum levels of ALP of Trpv6‐/‐ mice and WT mice. n = 5, **P < .01. (F) Histological sections of femurs stained for TRAP activity. (G) Quantitative analysis of TRAP‐stained area in femur sections of mice. n = 5, **P < .01. (H) Serum levels of CTX‐I of Trpv6‐/‐ mice and WT mice. n = 5, **P < .01

Abnormal bone formation or resorption leads to osteoporosis. Our results showed that the number of osteoblasts (N.Ob/BS) in *Trpv6^−/−^* mice was similar to that of WT mice, which was calculated using Masson's trichrome staining (Figure 2A,B). Similar results were attained for tetracycline double‐standard staining, and the mineral apposition rate (MAR) was comparable between the two genotypes (Figure 2C,D). In addition, there was no significant difference in serum ALP concentration between *Trpv6^−/−^* mice and WT mice (Figure 2E). TRAP staining in the bone sections further revealed the effect of Trpv6 depletion on osteoclastogenesis (Figure 2F). The number and surface area of osteoclasts in *Trpv6^−/−^* mice increased significantly (Figure 2G). Compared to WT mice, *Trpv6^−/−^* mice had higher levels of CTX‐1 (Figure 2H). To summarize, bone resorption was enhanced but bone formation remained unchanged in *Trpv6^−/−^* mice.

### 3.3 Trpv6 inhibits osteoclast formation via negative regulation of osteoclast differentiation and fusion

**FIGURE 3:**
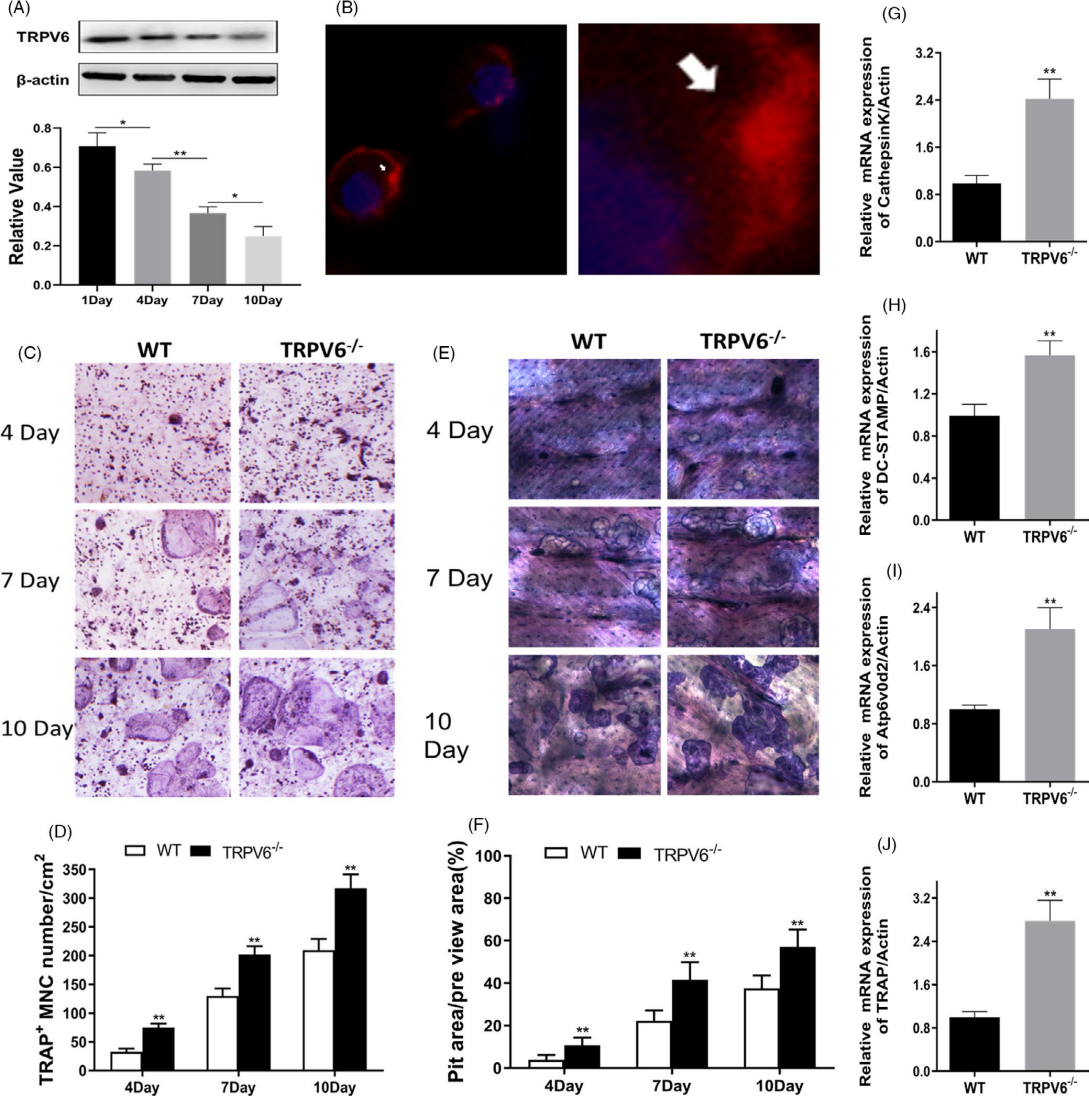
Trpv6 negatively regulates osteoclast differentiation and fusion and inhibits osteoclast formation. (A) Western blotting showed a time‐dependent decrease in Trpv6 expression during osteoclast differentiation. n = 3, *P < .05, **P < .01. (B) Confocal laser scanning microscopy showed Trpv6 staining (arrowhead) predominantly at the cell membrane. (C‐D) TRAP staining of osteoclasts precursors at different time points. Quantification of TRAP + multinucleated cells. Scale bars = 20μm. n = 5, **P < .01. (E‐F) Patterns of resorption pits on bovine cortical bone slices. Quantification of resorption area per view area. n = 3, **P < .01. (G‐J) qRT‐PCR analysis of the osteoclast formation‐specific genes Cathepsin K, DC‐STAMP, Atp6v0d2 and TRAP in each group under 50 ng/mL RANKL for seven days. n = 5, **P < .01

Western blotting suggested that Trpv6 was expressed in osteoclasts. The expression of Trpv6 in osteoclasts was reduced during cell differentiation (Figure 3A). Additionally, immunocytochemical fluorescence indicated that Trpv6 mainly localizes to the membrane of mature osteoclasts (Figure 3B). For deeper insight into the role of Trpv6 on osteoclast function, TRAP staining was employed. In cultures derived from *Trpv6^−/−^* mice, the number of TRAP^+^ multinucleated osteoclasts was significantly higher than in WT cultures (Figure 3C–D). A bone resorption lacuna experiment result showed that resorption pits were significantly enhanced in *Trpv6^−/−^* osteoclasts (Figure 3E–F). In addition, the mRNA levels of osteoclastogenesis marker genes (*cathepsin k*, *DC‐STAMP*, *Atp6v0d2* and *TRAP*) were obviously upregulated in Trpv6‐depleted osteoclasts (Figure 3G‐J).

### 3.4 Silencing of Trpv6 enhances osteoclast formation

**FIGURE 4:**
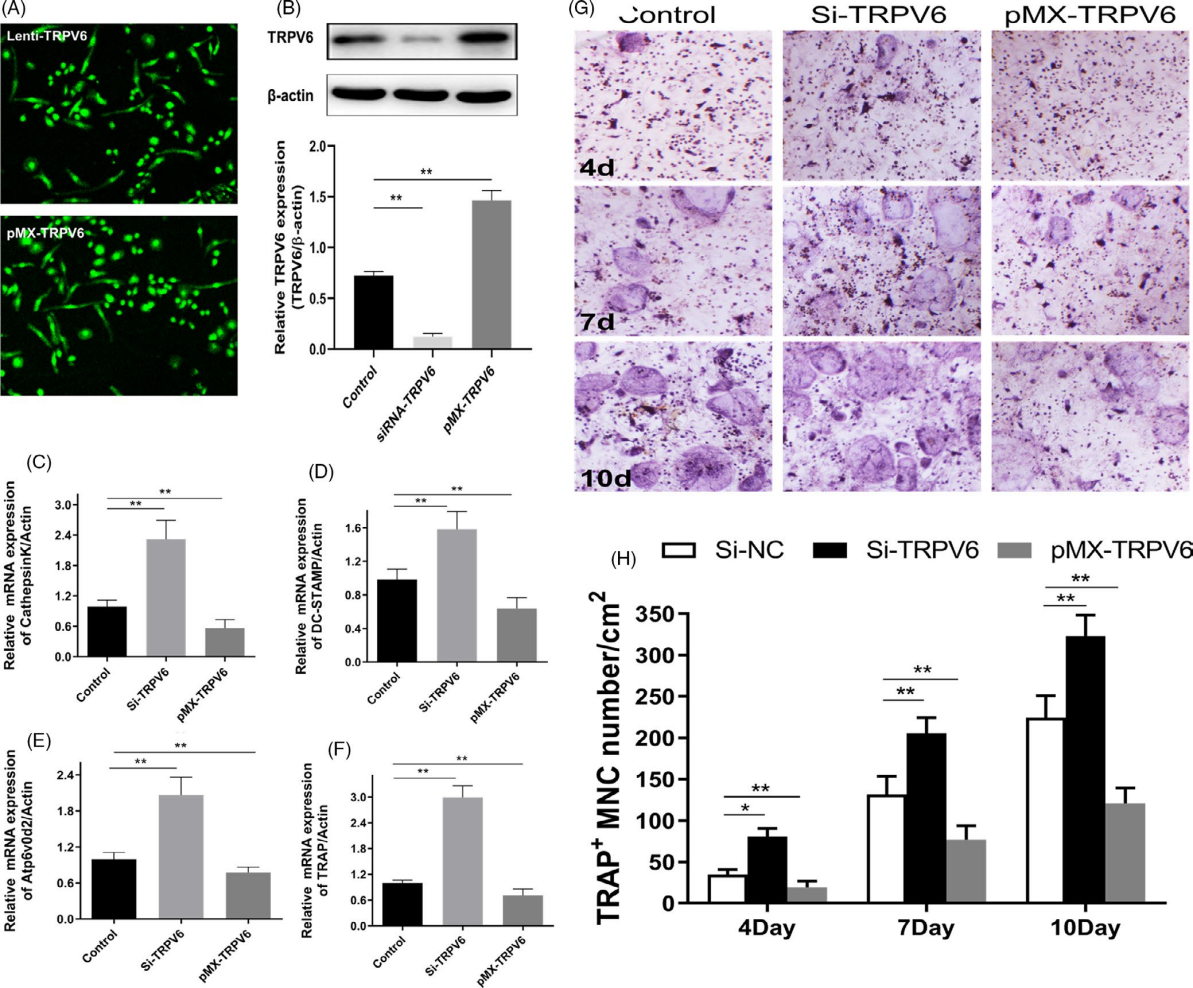
Silencing of Trpv6 enhanced osteoclast formation and bone resorption. (A) All cells expressed GFP, indicating that the cells were successfully infected by lentivirus. (B) Verified Trpv6 knockdown or overexpression by lentivirus‐mediated transduction of primary culture osteoclasts precursors. n = 5, **P < .01. (C‐F) qRT‐PCR analysis of the osteoclast formation‐specific genes Cathepsin K, DC‐STAMP, Atp6v0d2 and TRAP with Trpv6 knockdown or overexpression. n = 5, **P < .01. (G‐H) TRAP staining of osteoclasts precursors with Trpv6 knockdown or overexpression. Quantification of TRAP + multinucleated cells. Scale bars = 20μm. n = 5, *P < .05, **P < .01

To further verify the role of Trpv6 on osteoclast formation and bone resorption, we silenced and overexpressed *Trpv6* in osteoclasts by lentivirus transfection, respectively. Almost all cells expressed GFP, indicating that the lentivirus transfection rate was higher than 95% (Figure 4A). Western blotting confirmed that *Trpv6* was effectively silenced and overexpressed (Figure 4B). The mRNA levels of *cathepsin k*, *DC‐STAMP*, *Atp6v0d2* and *TRAP* were all elevated in osteoclasts infected with lenti–shRNA–TRPV6, whereas all were reduced in the group of osteoclasts infected with lenti–pMX–TRPV6 (Figure 4C–F). Next, we assessed the differentiation of osteoclasts with silenced or overexpressed *Trpv6* via TRAP staining assays. The number of stained multinuclear TRAP^+^ osteoclasts was obviously increased in osteoclasts with silenced *Trpv6* in a time‐dependent manner, whereas the Trpv6‐overexpressed group showed decreases (Figure 4G–h). The above results strongly suggested that Trpv6 acted as a negative regulator in osteoclast differentiation and function.

### 3.5 The IGF pathway is involved in the negative regulation of osteoclast formation and resorption by Trpv6

**FIGURE 5:**
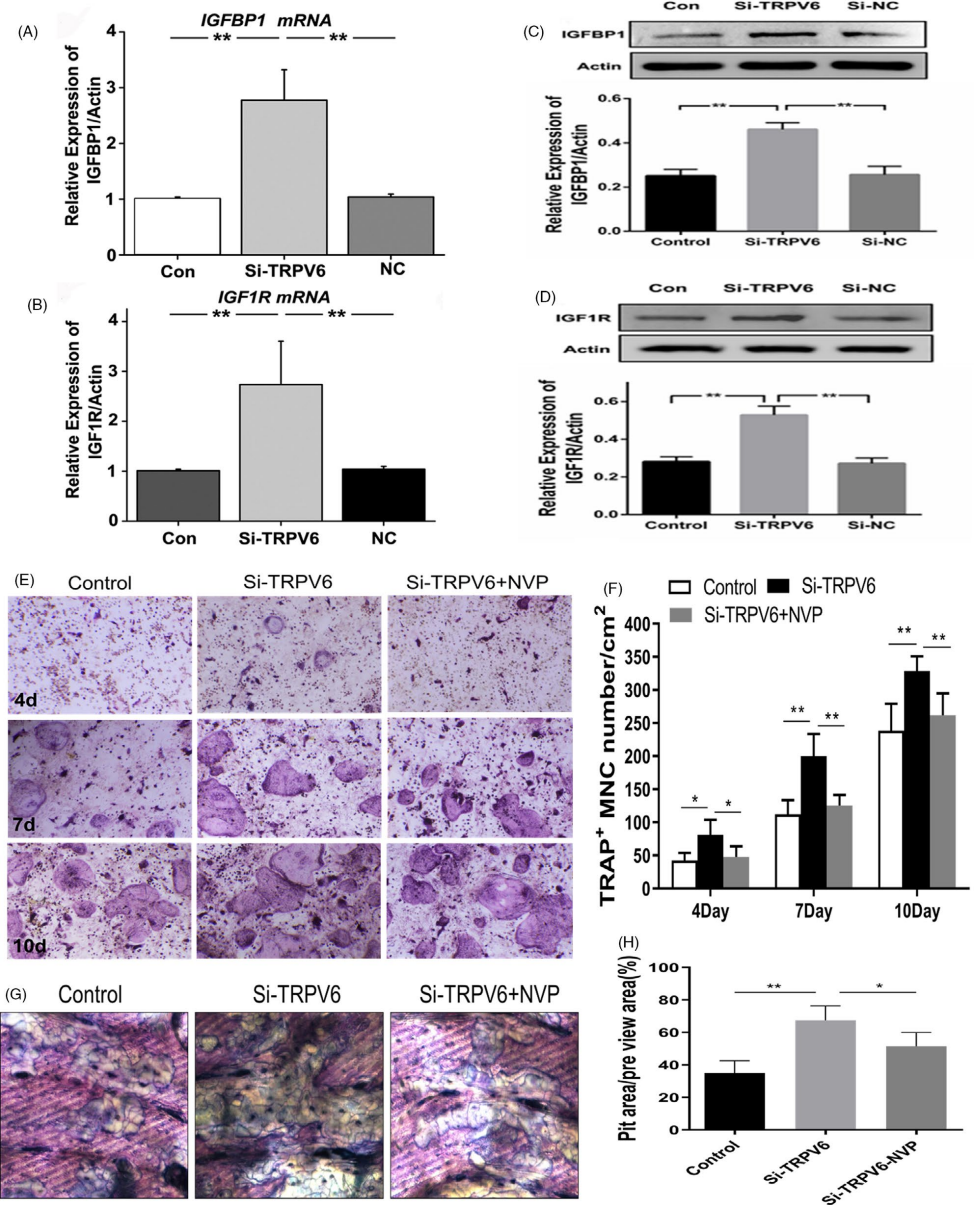
The IGF pathway was involved in the negative regulation of osteoclast formation and bone resorption by Trpv6. (A‐B) qRT‐PCR analysis showed that transcription levels of IGF1R and IGFBP1 in osteoclasts were significantly increased after Trpv6 silencing. n = 5, **P < .01. (C‐D) Western blot depicting that the protein abundance levels of IGF1R and IGFBP1 in osteoclasts were significantly increased after Trpv6 silencing. n = 5, **P < .01. (E‐F) TRAP staining revealed that adding the IGF1R antagonist NVP‐AEW541 to block the IGF signalling pathway significantly inhibited the osteoclast differentiation induced by Trpv6 silencing. n = 3, *P < .05, **P < .01. (G‐H) After the addition of the IGF1R blocker, the ability of bone resorption of osteoclasts induced by Trpv6 silencing was significantly inhibited, as evidenced by the bone resorption lacuna experiment. n = 3, *P < .05, **P < .01

Our previous study revealed that the RANKL‐induced [Ca^2+^] oscillation response was not significantly affected by inhibition of Trpv6.^16^ As a non‐Ca^2+^‐oscillating signalling pathway, the IGF pathway plays an important role in osteoclast formation.^28^, ^29^, ^30^ Therefore, we speculated that the IGF signalling pathway was potentially involved in the regulation of osteoclast differentiation and bone absorption by TRPV6. The results showed that the levels of IGF1R and IGFBP1 in osteoclasts were significantly increased upon silencing of *Trpv6* (Figure 5A–D), which confirmed our speculation. Next, we used the IGF1R antagonist NVP‐AEW541 to block the IGF signalling pathway. TRAP staining demonstrated that the inhibitory effect of Trpv6 on osteoclast differentiation was weakened in osteoclasts exposed to IGF blockers (Figure 5E,F). In accordance with the results of the TRAP staining, the IGF1R blocker significantly inhibited the induction of bone resorption in osteoclasts by silencing *Trpv6*, as evidenced by pit formation assays (Figure 5G,H). These results strongly suggested that Trpv6 negatively regulated osteoclast formation and bone resorption by inhibiting the IGF pathway.

### 3.6 Trpv6 negatively regulates osteoclast formation and bone resorption by inhibiting the IGF1R‐PI3K‐AKT pathway

**FIGURE 6:**
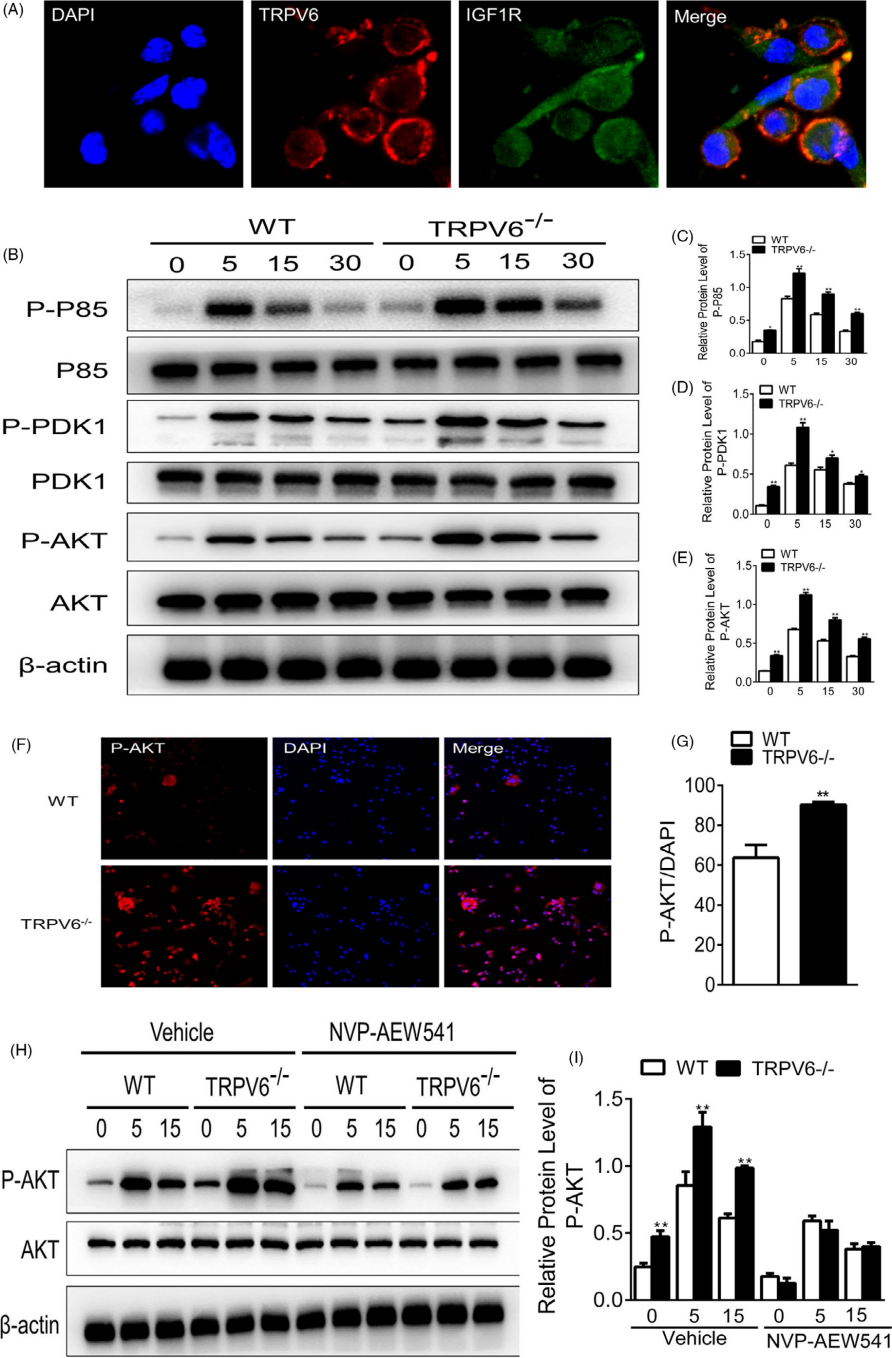
Trpv6 negatively regulates osteoclast formation and bone resorption by inhibiting the IGF1R‐PI3K‐AKT signalling pathway. (A) Immunofluorescence images of Trpv6 and IGF1R upon osteoclast induction with RANKL. Scale bars = 50μm. (B) At 0, 5, 15 and 30 min of treatment with RANKL, cells were harvested and analysed by Western blotting using anti‐P85, anti‐pP85, anti‐PDK1, anti‐pPDK1, anti‐AKT, anti‐pAKT and anti‐actin antibodies. (C‐E) Quantitative analysis showed that the ratio of p‐P85/P85, p‐PDK1/PDK and p‐AKT/AKT was increased in cells isolated from Trpv6−/− mice compared to WT mice. n = 5, *P < .05, **P < .01. (F‐G) Immunofluorescence of p‐AKT in osteoclast isolated from Trpv6‐/‐ mice and WT mice. n = 3, **P < .01. (H‐I) After NVP‐AEW541 blocking of the IGF signalling pathway, Western blotting revealed that there was no significant difference in the abundance ratio of p‐AKT/AKT between the osteoclast derived from TRPV6‐/‐ mice and WT mice. n = 5, *P < .05, **P < .01

The results of immunofluorescence results demonstrated that Trpv6 and IGF1R mainly localized to the membrane and cytoplasm of osteoclasts in (Figure 6A). To further explore the downstream signalling of the IGF pathway, BMMs isolated from *Trpv6^–/–^* and WT mice were induced by RANKL and M‐CSF for 7 days to form mature osteoclasts. The levels of phosphorylation markers p‐P85/P85, p‐PDK1/ PDK1 and p‐AKT/AKT in the PI3K–AKT pathway were then detected by Western blotting. The ratios of p‐P85/P85, p‐PDK1/ PDK1 and p‐AKT/AKT were increased in osteoclasts isolated from *Trpv6^–/–^* mice compared to WT mice (Figure 6B‐E). Consistent with these results, immunofluorescence staining showed that the ratio of p‐PDK1/PDK1 of osteoclasts isolated from *Trpv6^–/–^* mice was higher than that of osteoclasts isolated from WT mice (Figure 6F,G). Next, NVP–AEW541 was used to block the IGF signalling pathway and Western blotting revealed that there was no significant difference in the level of p‐AKT/AKT between the osteoclasts derived from Trpv6^–/–^ mice and WT mice (Figure 6H,I). The above results strongly suggested that Trpv6 negatively regulates osteoclast formation and bone resorption by inhibiting the IGF1R‐PI3K‐AKT pathway.

## 4 DISCUSSION

**FIGURE 7:**
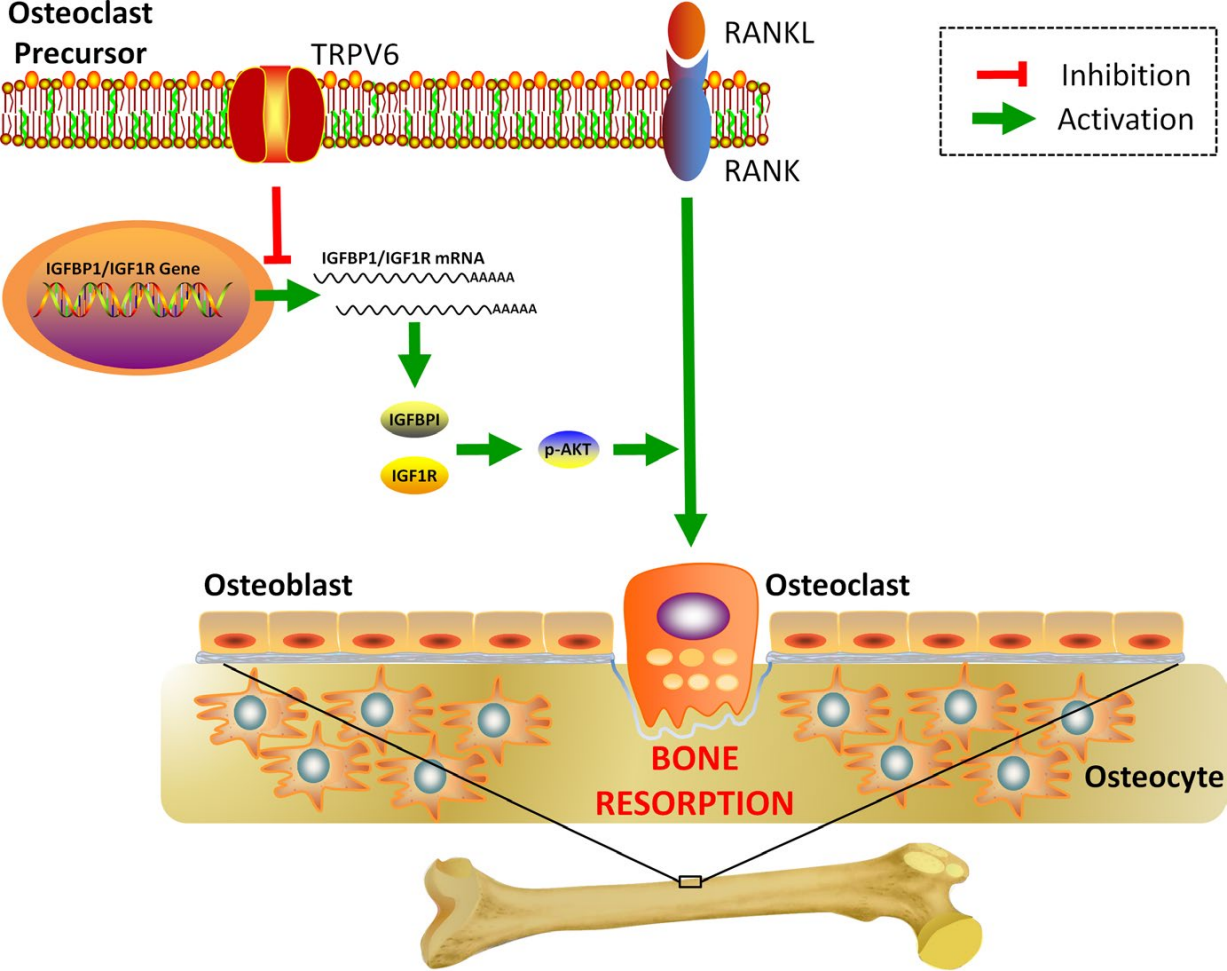
Illustrations of TRPV6 as a critical regulator in osteoclastic bone resorption. TRPV6 negatively regulates osteoclast formation and bone resorption by inhibiting the IGF/PI3K/AKT signalling pathway

In the present study, we demonstrated that TRPV6 was a critical negative regulator in RANKL‐induced osteoclast differentiation and bone resorption activity. Our studies for the first time demonstrated that TRPV6 decreased osteoclast formation and bone resorption through inhibition of the IGF–PI3K–AKT signalling pathway (Figure 7).

Bone metabolism maintains bone mass by balancing bone resorption and formation.^31^, ^32^, ^33^, ^34^ As a highly selective calcium channel in the TRPV subfamily, TRPV6 is an important protein in osteoclasts and participates in the regulation of osteoclast differentiation and bone resorption.^18^ Bianco et al^35^ found that *Trpv6^–/–^* mice had bone metabolism disorders, which manifested as intestinal calcium absorption dysfunction and decreased bone density. Lieben et al reported that *Trpv6^–/–^* mice fed with a low calcium diet exhibited significantly decreased bone mass, while bone resorption and bone formation were enhanced simultaneously, with bone resorption being more active than bone formation.^36^ In this study, we found that BMD, Tb.N and BV/TV were obviously decreased in Trpv6‐depleted mice, suggesting that TRPV6 is involved in bone metabolism. Furthermore, Masson's trichrome staining showed that N.Ob/BS was not decreased in *Trpv6^–/–^*. Similar results were revealed by tetracycline double‐standard staining, and MAR was comparable between the two groups. However, *Trpv6^–/–^* mice had significantly more TRAP^+^ cells in the metaphyseal region of femoral bone sections than WT mice. Therefore, based on the above findings, we hypothesized that Trpv6 contributes to bone homeostasis by regulating osteoclast differentiation and activity.

Previous studies have confirmed that osteoclasts arise from haematopoietic stem cells induced by RANKL and M‐CSF, which then undergo differentiation and fusion to form large multinucleated cells.^37^, ^38^ Several reports showed that TRPV6 regulated a variety of cellular functions by affecting intracellular Ca^2+^ concentrations, including differentiation, proliferation and apoptosis.^39^ The increasing concentration of Ca^2+^ in osteoclasts causes the cell pseudopodia to retract, restrict its movement, destroy the absorption skeleton structure and thus inhibit bone absorption.^40^ In our study, we found that *TRPV6* expression was decreased in a time‐dependent manner during osteoclast differentiation, and the distribution of TRPV6 in osteoclasts was mainly distributed across the cell membrane. In addition, TRAP staining and bone absorption pit experiments showed that the differentiation and fusion of osteoclasts isolated from *Trpv6^–/–^* mice were significantly quicker than that derived from WT mice. We also found that silencing *Trpv6* in osteoclasts significantly increased the rate of osteoclastogenesis. Thus, it is likely that the inhibitory role of TRPV6 in osteoclastic resorption is caused by limiting osteoclastogenesis.

Both independent signalling pathways and calcineurin‐dependent pathways contribute to NFATc1 activation, leading to efficient osteoclastogenesis.^41^, ^42^ Our previous research found that non‐Ca^2+^ oscillation signalling pathways contributed to TRPV6 deficiency‐induced osteoclastogenesis.^18^ As an important non‐Ca^2+^ oscillating pathway, the IGF signalling pathway is widely expressed in bone tissue and participates in the regulation of bone metabolism.^43^, ^44^ Dai et al^45^ found that TRPV5 negatively regulated the proliferation of NaR cells by inhibiting the IGF signalling pathway despite culture medium containing a normal calcium concentration. Our results showed that the silencing of *Trpv6* significantly increased the expression of two key proteins within the IGF signalling pathway, IGF1R and IGFBP1, in osteoclasts. NVP‐AEW541 is an effective and highly selective inhibitor of IGF‐1R/InsR.^46^ Blocking IGF1R with NVP‐AEW541 suppressed the increase of osteoclastic differentiation induced by inhibition of Trpv6. Taken together, we presume that TRPV6 is responsible for osteoclast formation and resorption through inhibition of the IGF signalling pathway.

As a downstream signalling pathway of the IGF signalling pathway, PI3K–AKT plays an important role in the activation of osteoclasts. AKT promotes osteoclast survival by regulating cell cytoskeletal replacement and movement, and knockdown of *AKT* reduces the expression of osteocalcin.^47^ Lee et al^48^ revealed that *PI3K*, *p38* and extracellular signal‐regulated kinase pathways were involved in osteoclast differentiation. Xing et al^49^ reported that the PI3K–AKT signalling pathway was involved in RANKL‐independent osteoclastogenesis. In the present study, Trpv6 inhibited the phosphorylation of PI3K–AKT in osteoclasts and reduced the abundance ratio of p‐P85/P85, p‐PDK1/PDK1 and p‐AKT/AKT. The effect of TRPV6 was eliminated after pre‐treatment with an IGF1R inhibitor (NVP‐AEW541).

In conclusion, our study confirmed the important role of TPRV6 in bone metabolism, clarified its regulatory role in osteoclast differentiation at the cellular level, and revealed the molecular mechanism of the negative regulation of osteoclastic formation and bone absorption by TRPV6. These results may provide a new strategy and a new target, TRPV6, for the treatment of osteoporosis.

## CONFLICT OF INTEREST

The authors declare that they have no conflict of interest.

## AUTHORS CONTRIBUTIONS

JM involved in conception and design, collection and assembly of data, data analysis and interpretation, and manuscript writing. LZ, ZBZ and TFX involved in collection and/ or assembly of data, and data analysis. LY analysed data. XY and AMC involved in conception and design, data analysis and interpretation, financial support and manuscript writing. TWY involved in conception and design, data analysis and interpretation, financial support, manuscript writing and final approval of manuscript. All authors read and approved the final manuscript.

## Data Availability

The data that support the findings of this study are available from the corresponding author upon reasonable request.
